# A Survey on Recent Trends and Open Issues in Energy Efficiency of 5G

**DOI:** 10.3390/s19143126

**Published:** 2019-07-15

**Authors:** Muhammad Usama, Melike Erol-Kantarci

**Affiliations:** School of Electrical Engineering and Computer Science, University of Ottawa, Ottawa, ON K1N 6N5, Canada

**Keywords:** 5G, energy-efficiency, sustainability

## Abstract

The rapidly increasing interest from various verticals for the upcoming 5th generation (5G) networks expect the network to support higher data rates and have an improved quality of service. This demand has been met so far by employing sophisticated transmission techniques including massive Multiple Input Multiple Output (MIMO), millimeter wave (mmWave) bands as well as bringing the computational power closer to the users via advanced baseband processing units at the base stations. Future evolution of the networks has also been assumed to open many new business horizons for the operators and the need of not only a resource efficient but also an energy efficient ecosystem has greatly been felt. The deployment of small cells has been envisioned as a promising answer for handling the massive heterogeneous traffic, but the adverse economic and environmental impacts cannot be neglected. Given that 10% of the world’s energy consumption is due to the Information and Communications Technology (ICT) industry, energy-efficiency has thus become one of the key performance indicators (KPI). Various avenues of optimization, game theory and machine learning have been investigated for enhancing power allocation for downlink and uplink channels, as well as other energy consumption/saving approaches. This paper surveys the recent works that address energy efficiency of the radio access as well as the core of wireless networks, and outlines related challenges and open issues.

## 1. Introduction

Advances in telecommunication systems around the world have always been pushing the wireless infrastructure to be more resilient and scalable. Ever growing faster data rates and a demand for the highest quality of service has been a strong constraint when energy conservation needs to be considered. Data rates as high as that of 1 Gbps have been foreseen with the advent of 5G. In addition, with an explosive number of heterogeneous devices coming online, including sensors for home security, tablets, and wearable health monitors, the computational power of base stations must increase. An estimated 50% increase in the computing power of baseband units has been predicted to handle this traffic burst [1]. Thus, the focus on energy-efficiency needs to include optimization of computational complexity in addition to optimization of transmission power.

An estimated 75% of the Information and Communications Technology (ICT) industry is supposed to be wireless by 2020 and today 5% of the world’s carbon footprint is coming from this industry alone. A consensus between academia and industry dictates that the foreseen 1000× capacity gain must be achieved with either the present energy consumption or lower [2]. Thanks to energy-efficiency efforts world-wide, energy consumption in the 5G realm, in terms of bits/joule, has been considered as an important design parameter. In 4th generation (4G), the concept of small cells has been introduced to increase the coverage and capacity. Therefore, [3] conducted an analysis on energy consumption per unit area for a heterogeneous deployment of cells for fourth generation networks. With 5G, small cells are inevitable in deployments due to their advantage of improved traffic handling within a smaller area as well as the shorter cell ranges that result from the use of higher frequencies. Yet, the increasing number of base stations translate into more energy consumption, although the increase in consumption will not be linear. Small cells, or in other words densification, calls for sophisticated management of resources. Most recently, intelligent resource allocation and control techniques utilizing machine learning algorithms have been suggested to help next generation radios in their autonomous reconfiguration for improving the data rates, energy efficiency and interference mitigation. Overall, the emerging sophistication in both User Equipment (UE) and network side has increased the energy consumption and thus objective functions have been devised to maximize the energy efficiency, harvested energy and energy aware transmission [4]. Many of the existing energy efficiency improvement techniques include the use of green energy sources for base stations, modifying the coverage area of a base station depending upon the load level, putting lightly loaded base stations to sleep and load balancing by handing over the UEs to the macro base station. A survey on these technologies for the 5G Radio Access Network (RAN) can be found in [5].

**Figure 1:**
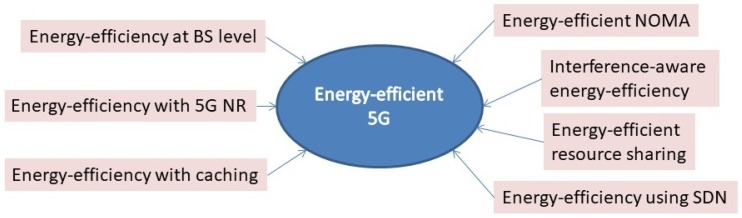
Outline of the energy-efficiency schemes included in this survey.

This survey has been aimed to contribute towards a greener and a sustainable telecommunication’s ecosystem by reviewing and bringing together some of the latest ideas and techniques of energy conservation at base station and network level. A high level diagram shows the areas addressed in Figure 1. A few of the prominent examples include the introduction of a newer Radio Resource Control (RRC) state for context signalling and cutting down on the redundant state changes [6]. Utilization of advanced clustering and caching techniques on the RAN side have been highly appreciated for their benefits of improving the latency of getting the data requested by a group of users and possibly eliminating the factor of clogging the network by a huge number of requests for the same content [7,8]. A case study of commercial resource sharing among different operators bears fruitful results in terms of reduced deployment costs and good data rates with minimum interference among them [9]. The upcoming sections introduce the basics of energy efficiency, provide justification for the need of gauging the energy consumption and then present the most recent research works carried out for the optimization at different levels of the architecture. This survey bears its uniqueness in its holistic approach to energy-efficiency by covering radio, core and computing side of 5G. This paper is also different than the surveys in the literature [1,2,3,4], as it focuses on works published in the last few years where the majority of the studies focus on concepts specific to the new 5G standard.

## 2. Background on Energy Efficiency

A formal relationship between energy efficiency and Signal to Interference Noise Ratio (SINR) has been presented in [2] using the bit/joule notion. Meanwhile, Reference [4] lays the foundation for energy efficiency in different parts of the network including base stations and the core network. In the literature, energy saving and use of green energy resources have been the two mainstream approaches to offer energy efficiency. Among the energy saving techniques, cell-switch off techniques have been widely exploited. For instance, in the EU FP7 ABSOLUTE project, an energy aware middleware has been proposed that would use the capacity-based thresholds for activation of the base stations [10]. In several other studies, data offloading has been considered as an energy-efficient approach. Furthermore, authors in [11] have put together several techniques for not only reducing the energy consumption from the traditional energy sources but also for surveying newer Energy Efficiency (EE) schemes in the End-to-End (E2E) system. One of the remarkable mentions by the authors includes the implementation of 3rd Generation Partnership Project (3GPP) compliant EE manager that would be responsible for monitoring energy demands in an E2E session and for implementation of the policies needed for catering to the ongoing energy demand.

In addition to energy saving approaches, recently simultaneous wireless energy transfer has been studied. Furthermore, local caching techniques have been proved to be beneficial for relieving the load on the backhaul network by storing the content locally and limiting the re-transmissions, hence reducing energy consumption. Similarly, a cloud based RAN has been envisioned as a possible solution for the computational redistribution in [2,4,12]. Many of the tasks previously performed by a base station (BS) would be taken away to a data center and only decision making for Radio Frequency (RF) chains as well as baseband to RF conversion would be given to base stations. Traffic pattern and demands would then be catered for well before time and redundant BS would be put to sleep mode according to [13]. Furthermore, full duplex Device-to-Device (D2D) communication with uplink channel reuse has been considered to improve SINR and transmission power constraints. A gain of 36% energy efficiency has been demonstrated using the full duplex scheme with enhanced self-interference mitigation mechanism instead of half duplex [14].

As machine learning is penetrating more and more into the operation of wireless networks, Reference [15] suggests that machine learning algorithms would greatly help to predict the hot spots so that other resources could be switched off when not needed.

The concept of energy efficiency being treated as a key performance indicator in the upcoming 5G standard considers it to be a global ambition, but it cannot be declared as a specific actionable item on either the operator or vendor side. Divide and conquer approach has been applied to the entire network and improvements have been targeted at either component level, equipment level or at network level employing newer algorithms at both BS and UE side. This discussion advocates the fact that operators would have the leverage of tuning their network for a balance between quality of service and energy consumption. In the following sections, we introduce the recent works in energy-efficiency in 5G as highlighted in Table 1 preceding to a discussion on open issues and challenges.

## 3. Review of EE Techniques at the Base Station Level

Radio access network (RAN) has been considered as single unit for energy efficiency improvement, and inclusion of these enhancements across the network would have a significant impact on the overall energy efficiency. Metrics for gauging EE in this perspective include the improvements in the architecture and chipset design for the baseband units, cell switch off techniques, incorporation of small cells, interference reduction among the neighboring cells and caching as well as the newer RRC state for UEs for conservation of the battery power.

### 3.1. Base Station Energy Consumption and Cell Switch Off Techniques

**Figure 2:**
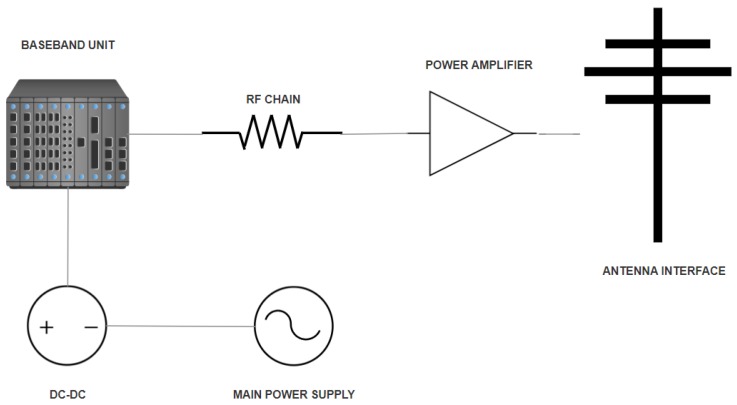
Modules of a typical base station.

Knowing the accurate energy consumption of a base station constitutes an important part of the understanding of the energy budget of a wireless network. For this purpose, authors in [1] have specifically discussed energy conservation at equipment level by presenting the breakdown of a base station. A typical BS has been presented by dividing it into five parts, namely antenna interface, power amplifier, RF chains, Baseband unit, mains power supply and the DC-DC supply. These modules have been shown in Figure 2. An important claim has been made stating that up to 57% of the power consumption at a base station is experienced at the transmission end, i.e., the power amplifier and antenna interface. Yet, with small cells, the power consumption per base station has been reduced due to shorter distances between the base stations and the users [1,19]. In [19], analytical modelling of the energy efficiency for a heterogeneous network comprising upon macro, pico and femto base stations has been discussed. To a certain extent emphasis has been put on the baseband unit which is specifically in charge of the computing operations and must be sophisticated enough to handle huge bursts of traffic. A baseband unit has been described to be composed of four different logical systems including a baseband system used for evaluating Fast Fourier Transforms (FFT) and wireless channel coding, the control system for resource allocation, the transfer system used for management operations among neighbouring base stations and finally the system for powering up the entire base station site including cooling and monitoring systems. Furthermore, the use of mmWave and massive MIMO would need an even greater push on the computation side of the base station since more and more users are now being accommodated. The study in [16] discusses the achievable sum rates and energy efficiency of a downlink single cell M-MIMO systems under various precoding schemes whereas several design constraints and future opportunities concerning existing and upcoming MIMO technologies have been discussed in [17]. The computation power of base station would increase when number of antennas and the bandwidth increases. In the case of using 128 antennas the computation power would go as high as 3000 W for a macrocell and 800 W for a small cell according to [1].

Authors in [18] have discussed the utility of taking most of the baseband processing functionality away from the base station towards a central, more powerful and organized unit for supporting higher data rates and traffic density. Users have envisioned experiencing more flexibility using this central RAN since they would be able to get signaling from one BS and get data transfer through another best possible neighboring BS. Visible gains in latency and fronthaul bandwidth have thus been observed by having stronger backhaul links but this research avenue still needs to be formally exploited for devising globally energy efficient mechanisms. The choice of the best suited BS would allow the network to have a lower transmission power thus increasing the energy efficiency. An analysis of throughput as a performance metric has been provided for a two-tier heterogeneous network comprising upon macro and femto cells in [20]. The claimed improvement in throughput originates from a distributed mesh of small cells so that the minimal transmission distance between the end user and the serving base station would be cashed out in terms of reduced antenna’s transmission power. Considering these findings on BS energy consumption, cell switch-off techniques have been explored in the literature. An incentive based sleeping mechanism for densely deployed femtocells has been considered in [21] and energy consumption reduction up to 40% has been observed by turning the RF chains off and only keeping the backhaul links alive. The key enabler here would be to have prompt toggling between active and sleep modes for maintaining the quality of service. According to [21], a “sniffer” component installed at these small cells that would be responsible for detecting activity in the network by checking the power in uplink connections, a value surpassing the threshold, would indicate a connection with the macrocell. Mobility Management Entity (MME) has also been suggested to potentially take a lead by sending wake up signals to the respective femtocells and keeping others asleep. In contrast to the usual techniques of handing their users over to the neighbouring base stations and turning that cell off, it would be beneficial to give incentives to users for connecting to a neighbouring cell if they get to have better data rates. Authors in [22] have conducted a thorough study for classification of the switching techniques as well as calculation of the outage probability of UEs, under realistic constraints. Their claim states that the energy consumption of the base station is not directly proportional to its load so an improved switching algorithm was needed that would allow the UEs to maintain the SINR thresholds. They have thus brought forward a sector based switching technique for the first time. Furthermore, their claim favors an offline switching technique instead of a more dynamic online scheme because of practical constraints such as random UE distribution and realistic interference modelling. Authors in [23] discuss influence of the transmit power scaling and on/off switching on instantaneous macro base stations power consumption. The proposed power consumption models have been claimed to be used as generic models for the relationship between transmitted and consumed power for macro base stations of different technologies and generations. In addition to these techniques, recently, machine learning techniques have been used to implement cell switch off which are discussed in Section 6.

### 3.2. Interference-Aware Energy Efficiency Techniques in 5G Ultra Dense Networks

The advantages of small cell deployment, in terms of increased system capacity and better load balancing capability, have been discussed in the previous sections. Yet, it is important to mention that densification suffers from added system complexity. Therefore, energy efficiency as well as spectral efficiency becomes harder to evaluate. Nash energy efficiency maximization theory has been presented for discussing the relationship between energy and spectral efficiency in [24]. Both are inversely related to each other, increase in one of them demands a natural decrease in the other quantity which usually has been the case of medium to high transmission power. Most of the research conducted in ultra-dense small cell networks has been on coming up with techniques optimizing both energy efficiency (EE) and spectral efficiency (SE). Authors in [24] also brings forth the idea of gaining energy efficiency at the cost of spectral efficiency where the small cells are under the coverage of a macro cell and pose interference issues due to the sharing of bandwidth among them.In such a scenario, all the small cells participate in energy efficiency maximization according to a game theoretic methodology. The suggested game theoretic model has been deemed to be a distributed model and utilizes Nash product for maximizing cooperative energy efficiency. Analysis of the algorithms shows that energy efficiency, although it increases with the increase in the number of small cells, it saturates after about 200 cells and afterwards only experiences a minor increase. Fractional programming has been extensively used in [25] for modelling the energy efficiency ratio for a Point-to-Point (P2P) network as well as for a full scaled communication network using MIMO. EE has been considered as a cost benefit ratio and minimum rate constraints have been put together for modelling real life scenarios. In addition, fairness in resource allocation has been considered a major factor in the overall energy distribution. These two constraints might tend to increase the power consumption in case the minimum thresholds tend to be too high. Adding to the use cases of fractional programming, [26] laid out a robust distributed algorithm for reducing the adverse effects of computational complexity and noise towards resource allocation. Authors in [27], have presented an experimental setup for defining the right kind of key performance indicators when measuring either EE or SE. The setup includes a set of UE(s), three small BS(s) and running iperf traffic using User Datagram Protocol (UDP) and File Transfer Protocol (FTP). Results have indicated that utilization of a higher bandwidth would not increase the power consumption, that throughput must incorporate the traffic density and that the idle power of the equipment needs to be considered for energy consumption calculations. In [28], use of varying transmission power levels by the aid of custom power levels in a two-tier network has been encouraged for the optimization of needed power in Long Term Evolution (LTE). Intelligent switching of control channels in the DL and tuning the power levels according to the UE’s feedback have been envisioned to aid in allocation of the resource blocks with an optimum power. Authors in [29], have discussed the opportunities for the less explored domain of user scheduling in LTE. 3GPP has no fixed requirement on scheduling and thus researchers have devised their own mechanisms depending upon their pain points. Authors have proposed the idea of associating Quality of Service (QoS) with scheduling for accommodating cell edge users. Authors in [30] have proposed a resource allocation technique for minimizing the interference at the UE side. Considering a full duplex communication setup, a circular interference area for a DL UE has been demarcated by the BS based upon a predefined threshold. Resource block for this UE has been shared by an UL UE from outside the interference region for keeping the mutual interference to a minimal level. Simulation results claim to improve the overall network throughput based on the efficient pairing of UEs but the throughput might degrade with a large increase in the distance between the paired UEs. A heuristic algorithm presented in [31] improves the system throughput using resource reuse in the three-tier architecture while regulating the interference regions of UEs being served by either macro BS, small BS or in a D2D way. Visible gains in the throughput have been noted with an increased user density for an efficient user selection and having a minimum distance between the UEs being served in a D2D fashion for a stronger link retention. Moreover in [32], authors have constructed objective functions for EE maximization and have thus compared max-min power consumption model against their nonlinear fractional optimization model. Results have been promising for a reduction in the power consumption because of the mutual participation of cells as their number starts to increase.

### 3.3. Energy Efficiency Enhancement with RRC Connection Control for 5G New Radio (NR)

**Figure 3:**
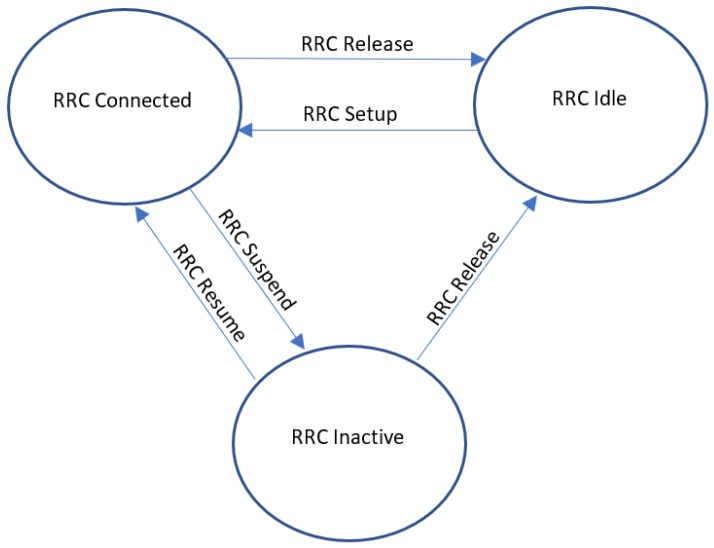
State diagram for radio resource control (RRC) signalling including the ’inactive’ state.

In [6], the authors discuss the rapid UE battery drainage which is due to the fact that terminals remain in radio resource control’s (RRC) ACTIVE state even when they are not interacting with the network. In the 5G networks, the RRC INACTIVE state has greatly been altered where a UE could benefit from the stored context and go through a lower number of state transitions. 5G NR would thus get rid of the constant monitoring of physical downlink control channel (PDCCH) for the incoming transmissions. The proposed improvement brings a 50% less energy consumption at the modem and 18% for the entire device. Referring to the traditional RRC mechanism, only two states were available, namely RRC ACTIVE and RRC IDLE mode. Consumer’s usage mainly dictates the time being spent in either of the two states. Typically, when a phone has not been used, the user inactivity timer would expire, putting the UE in IDLE state and as soon as it would go into the IDLE state its context would be removed from the core network. With the new RRC INACTIVE state, the UE context would still be stored when it would stop its communication with the network resulting in a reduced signaling overhead. However, the UE would still need to update eNodeB/gNodeB (evolved NodeB/next generation evolved NodeB) with its context for a valid state change. Figure 3 illustrates the state diagram of the new model. For this state to be widely utilized it should ensure minimum signaling and power consumption. The authors have evaluated the performance of this proposed scheme based on the shorter user inactivity timer achieving quicker state transitions to INACTIVE state and incurring less signaling. Power consumption analysis has been conducted for usage between different applications which validates the claim of authors. Similar analyses have been conducted to eliminate the prolonged connected mode discontinuous reception or better known as the Connected mode DRX (C-DRX) of upto 10 s for short data transfers and avoid the state changes. Signaling overhead also increases with the increase in either UE mobility or shorter user activity timers. However, the worst-case scenario would be to have the UE receive content just after its transition to the INACTIVE state, thus incurring extra RRC signaling. According to the proposed scheme, 5G NR can greatly benefit from this state by having an extended UE life and a lower need for S1 signaling.

### 3.4. Energy Efficient and Cache-Enabled 5G

**Figure 4:**
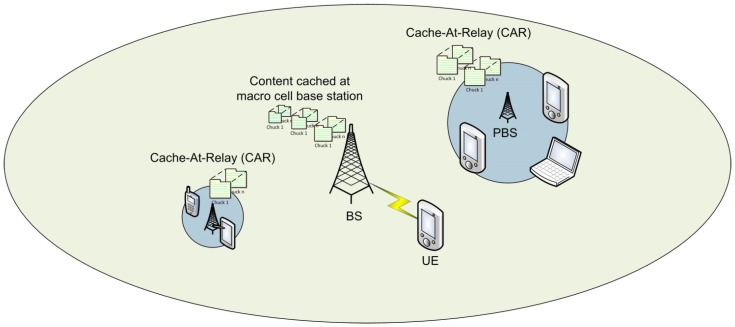
Illustration of different cache topologies.

In [7], the idea of proactive caching based on the content popularity on small cells has been proposed for improving the energy efficiency. Owing to the abundance of small cells, networks are getting constrained by the overall backhaul link capacity and much of the load is corresponding to transactions of the same requests repeatedly. Energy efficiency has been evaluated with regards to the content placement techniques and more emphasis has been put into organizing the content based on user locations and constantly fine tuning the clusters based on the content popularity distribution instead of spanning the same content across the network. Various topologies are shown in Figure 4. Energy efficiency has been formulated in relation to the small cell density vector. A heterogeneous file popularity distribution has been considered and a popularity vector has been maintained at every user. Users have been grouped into clusters depending upon the similarity in their interests and the cached files are an average of these popularity vectors. Users would usually be allowed to communicate with the base station within a specified distance of their cluster and in case of a cache miss event, the content would then be requested from the core via backhaul links. Spanning the same data across the network tends to sacrifice the information diversity and hence a content-based clustering approach has been brought forward. Simulations have been presented to demonstrate that with the increased base station density, significant energy efficiency gains have been experienced since the allocation problem gets simplified and interference and transmission powers would be reduced. In [34] a unique approach for addressing the energy efficiency challenge has been presented. The proposed E3 ratio thus incorporates a cost factor when calculating the number of UEs being served against the power spent over this operation by the BS. It has been made clear that although the cost factor might not have a direct impact on the spectral efficiency, it would be an important factor when regulating the cost of the entire network. Thus, operators have been addressed to carefully incorporate the features of edge caching and gigabit X-haul links to strike a fair balance between the cost overhead and the need of the feature. Otherwise it would be an overkill which has been meant to be strictly avoided. Mathematical analysis for EE maximization presented in [35] supports the fact that for the cases of low user cache size, non coded schemes should be utilized for a faster delivery system. Highlight of the research work conducted in [33] has been the assumption of a finite cache memory for a more realistic analysis. Delay bounds of an online cooperative caching scheme have been brought forward as compared to offline and a random caching scheme. The cache being periodically updated promises to deliver a tighter user association and aims to have minimum possible latency. The algorithm also aims to accurately cache the data in highest demand with an increased user density. Application of cooperative caching on P2P networks has been discussed in [37], authors have demonstrated the effectiveness of the algorithm by the segmentation of cache memory at the base stations. It would not only keep track of the cached data of the highly demanded information but would also record data paths and the newly requested data. The simulations have illustrated the usefulness of this optimization technique by the reduced number of hops and latency. On the other hand, uplink energy conservation has been considered in the context of dense small cells [36].

In [8], energy efficiency analysis of heterogeneous cache enabled 5G hyper cellular networks was performed. The control and user plane separation is considered to aid in devising enhanced access schemes and retain fairness in service. Furthermore, base station on-off strategy is taken into account to help in cutting down costs spent on redundant small cells [8]. In that scenario, macro cells would be the masters handling mobility, home subscriber and the user admission whereas small cells would be the slave part of the radio resource management scheme. With this increasing growth of the network infrastructure, irregularities in traffic behavior must be taken into account along with the actual user distribution for a realistic scenario. Caching has been sought after as a viable solution for reducing the end to end latency by storing content at the base stations. Small cells would typically involve macro base station in its communication with the UE in a semi sleep mode and ensure that it would always be aware of the UE positioning in the network as well as the cache memory statistics. Macro cell also ensures that the UE would be served by the closest and best possible small cell and would turn off the remaining ones to concentrate on a specified area for improving the throughput. On the other hand, there would be a predefined search radius and content would be fetched from a neighbouring base station within that distance. Otherwise, UE would associate to the macro base station for getting access to the needed content. Expressions for the coverage probability for the UE to get signal to interference (SIR) ratio within the threshold, throughput and power consumption and efficiency have been documented in [8].

## 4. Review of EE Techniques at the Network Level

A collective approach has been adopted for addressing the overall EE challenge considering both access and core network. EE has thus been gauged by the extent of resource sharing among different operators in the urban environment, utilization of efficient resource allocation schemes for fully exploiting the available spectrum, deploying middle ware for coverage enhancement (reduction in the distance between UE and BS would lower the needed transmission power), harnessing maximum computational muscle for accommodating massive incoming user requests yet have the ability to scale instantly (virtualization) and deploying machine learning and Software Defined Network (SDN) technologies for a fine grained control over the resources. An efficient usage of these capabilities would thus lead to the quality of service retention as well as an excellent power management methodology.

### 4.1. Resource Sharing in 5G with Energy-Efficiency Goal

Spectrum and physical resource sharing needs to be considered for accomplishing the energy efficiency goal of 5G. However, the need of service quality retention with respect to throughput and packet drops must also be addressed. Thoughts on infrastructure sharing have been gaining enough traction owing to several factors, for example, lack of space acquisition for site deployment or utilizing the available resources at their full potential and refraining from any new deployment. This section puts together the studies for bringing improvements in energy efficiency by a mutual sharing of infrastructure. Operators would have the flexibility of resource sharing at either full or partial level naturally emphasizing improved security for their equipment. Additionally, the cost of commissioning every site would lead to a higher expenditure and would minimize the expected revenues. Projects such as EARTH and GREEN TOUCH detail this avenue and brings forth an expectation of a decreased energy consumption by 1000 folds [2,38]. For this level of sophisticated resource sharing, a complete knowledge about the functionality and capacity of the network entities needs to be available which may not be possible in practice. However, the avenue of spectrum sharing still welcomes more discussion and aims to be a potential pathway for gaining solutions to the resource scarcity problem. Details of system level simulations for comparisons drawn between energy consumption and shared infrastructure at different load levels have been documented in [38] where a gain of up to 55% for energy efficiency in the dense areas has been demonstrated. Other significant advantages of resource sharing would include less interference by a planned cell deployment in accordance with the user demands per area. These efforts aim to eliminate the problems of either over provisioning or under-utilization of the deployed network entities. Authors in [40] have discussed the application of an improved resource allocation in a fog RAN. The suggested idea relies upon the fact that the usage of a centralized baseband processing unit, which, while increasing the processing power of the system, remains at risk of getting outdated measurements from the radio heads because of larger transport delays. The suggested algorithm starts off by switching off the redundant access points for conserving the energy and then modifying the beam weights for providing the end user with an optimum signal to leakage and noise ratio. User association is made centrally and then the information gets passed on to the fog access points after being scheduled for users. Following this phase, the proposed greedy algorithm tracks the global as well as the local energy efficiency readings and switches off the access points not needed until the rising trend of global energy efficiency ceases. Simulations have been carried out using a layout of macro and pico cells showing about a three-fold increase in the reported Channel State Information (CSI). Furthermore, authors in [39] have demonstrated the EE gains in a dynamic six-sector BS, capable of operating at either one or a maximum of all the sectors fully functioning, to be up to 75% as compared to the case of an always on approach.

In [9], a case study of infrastructure sharing between different operators has been presented as well. Service level agreement between the participating operators is defined and handled by multi-objective optimization methods. In such a shared environment, QoS should go hand in hand with fair resource utilization. Authors have specifically considered the case of obeying operator specific energy and spectral efficiency criteria along with the global spectral and energy efficiency maximization. The most prominent outcomes of this research are the global energy and spectral efficiency maximization in a shared noise-limited environment and the application of the framework to a network shared by any number of operators each serving different numbers of users and an optimal fulfillment of utility targets. Detailed mathematical analysis has been presented for system modelling with noise and interference constraints. SINR equations, which originally were used as a starting point, were thus gradually modified by incorporating weighting factors for influencing the priorities. This model turns out to be working in a polynomial complexity and maximizes the given objective function. Moreover, maximum and minimum bounds have been enclosed. In the paper, authors have presented the application of the mathematical tools by presenting the case of a base station installed in a crowded place such as an airport or shopping mall where the site owner is the neutral party and the frequency resources are either pooled or one of the operators grants some of his portion to others. Firstly, the case of two operators has been presented when they do not have any global constraints and the multi-objective problem set of noise limited scenario would be used. Secondly, site owner restricts the interference level or the global energy efficiency for both the operators and both of them target a minimum QoS constraint. Thirdly, there would be three operators with the same condition as of the first case. The work has laid the foundation to establish the criterion for the energy-spectral trade off in a single/multi carrier scenario.

### 4.2. Energy Efficient Resource Allocation in NOMA

**Figure 5:**
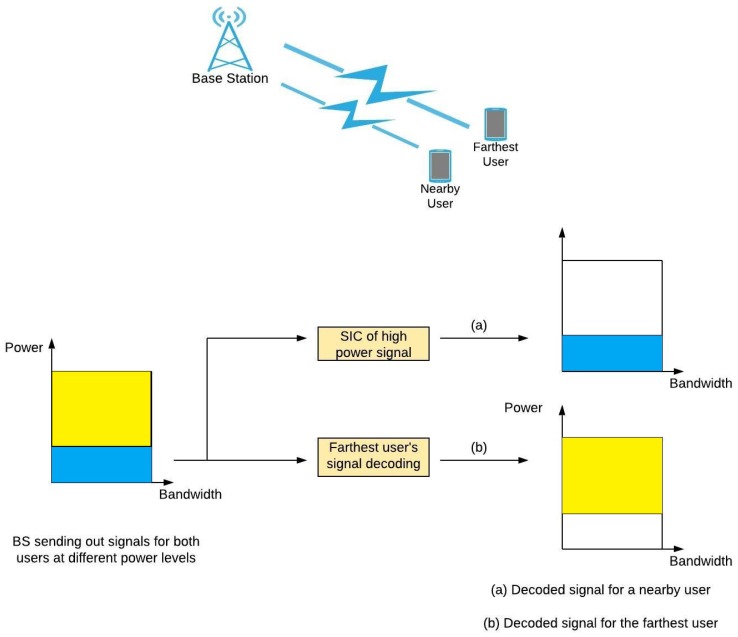
Concept of non-orthogonal multiple access (NOMA) technology.

In 5G, attempts have been made to possibly explore the area of non-orthogonal multiple access (NOMA), employing power control for saving resources in both time and frequency domain. This concept is highlighted in the following Figure 5. Operators would benefit from this technique by getting to serve the maximum number of users within the same frequency band, thus improving spectral efficiency [41]. This research area has been active for a while now for the reasons of increasing the network capacity and improving the data rates. An intelligent coordination among the base stations must be in place for maximum utilization of the available overall network energy. This corresponds to the fact that the harvested green energy has mostly been volatile, and a constant input source could not be guaranteed. For this reason, a detailed mathematical model has been presented for the power control of the UEs being serviced for minimizing interference as much as possible. A comparison of user association based genetic algorithms against a fixed transmit power was drawn. NOMA based techniques were demonstrated to outperform the conventional techniques for EE improvement for a larger number of nodes. The application was extended to a two-tier RAN having a macro base station covering a region of several pico base stations, being powered by both green and conventional energy sources. The proposed mathematical model uses a ratio of the network’s data rate over the entire energy consumption as the network utility. Incorporation of improved user association techniques were suggested in [42] for improvement of user throughput and error containment in NOMA. In [43], authors presented the mathematical feasibility for the utilization of successive interference cancellation at the receiver side. The signal that is being processed considers others to be noise, cancels them out and its iterative nature aims to decode all of them. With an increase in the number of transmitters having a fixed SINR, a linear relationship has been observed. On the other hand, this formulation might lead to a saturation point for the explosive number of IoT devices.

The authors in [44], have taken an interesting approach for a fair comparison of NOMA and a relay-aided multiple access (RAMA) technique and a simulation was carried out for maximization of the sum rate. It was established via mathematical formulation that sum rate is an increasing function of user’s transmission power and for the cases of a high data rate demand of the farthest user, NOMA proved to have maximized the sum rate. Distance between the users has been a key figure and with an increased separation between them, NOMA provides maximum rates whereas for the smaller separation relay-based setup provides a good enough sum rate. Authors in [45] have endorsed the advantages of nonorthogonal multiple access (NOMA) for the future radio access networks. Apart from the fact that the technique aids in getting a better spectral efficiency, authors instead have analyzed the feasibility of acquiring a better energy efficiency out of it as well. Considering the example of one base station serving two users, relationships between SE and EE have been observed which reflects that NOMA can potentially regulate the energy within the network by the allocation of more bandwidth to a cell center user in the uplink and more power to the cell edge user in the downlink. Considering the potential of NOMA, the problem was tackled with respect to its deployment scenario for the maximum exploitation. For a single cell deployment, EE mapping against resource allocation was considered as an NP hard problem because each user would be competing for the same radio resource, however, user scheduling and multiple access methods would aid for improving this situation. For the network level NOMA, a joint transmission technique could be beneficial for organizing the traffic load on the radio links and users must be scheduled accordingly when it comes to energy harvesting to keep the users with critical needs prioritized. Lastly, Grant free transmission has been studied for saving the signaling overhead, as soon as the user acquires data in its buffer it should start the uplink transmission and selection of the received data would be based upon its unique multiple access signature. Multiple access signature is deemed to be the basis of this proposal, but the signature pool must be carefully devised with an optimal tradeoff between the pool size and mutual correlation. It would greatly help for collision avoidance and detection. The users remain inactive for cutting down on the grant signaling and hence more energy is typically conserved. The proposed hybrid technique transitions between grant free and scheduled NOMA based on the current traffic load which eventually lowers down the collision probability and improves latency. In contrast with the above works that have discussed the use cases of caching in orthogonal multiple access (OMA), authors in [46] explored index based chaching instead of superposition chaching while adopting a sub optimal user clustering technique for significant reductions in the transmitted power while using NOMA. Owing to the enormous number of users, optimal user clustering was discouraged and user association based upon their differences in terms of link gain and cached data was suggested instead. The iterative power allocation algorithm was demonstrated to converge after several iterations.

### 4.3. Energy Efficient 5G Outdoor-Indoor Communication

The research in [47] discusses a use case of shared UE side distributed antenna system for indoor usage where a combination of distributed antenna and MIMO technology is used for getting enhancements in the coverage area and utilization of unlicensed frequencies for accommodating more users. The use of both licensed as well as unlicensed bands simultaneously needs a redesign of the current resource allocation algorithms [47]. In this work, resource allocation has been considered to be a non-convex optimization for increasing the end to end energy efficiency. The suggested topology demands installation of a shared UE side multiple antenna hardware between a single antenna base station (outdoor) and arbitrary number of single antenna UEs (indoor) which are called shared user equipment (UE)-side distributed antenna system (SUDACs). These SUDACs would be able to communicate the channel information with their neighbouring SUDAC units installed. In contrast with the relaying in the LTE-A system, SUDACs could be installed at different locations by the users and still be able to operate in both licensed and unlicensed bands simultaneously. The problem statement boils down to defining the energy efficiency in terms of the bits exchanged between base station and the UEs via SUDACs per joule of energy. It has been shown in [47] that application of this model exploits the frequency and spatial multiplexing of UEs and increases the system efficiency as compared to the case when SUDACs is not involved.

### 4.4. Energy Efficient Virtualization in 5G

**Figure 6:**
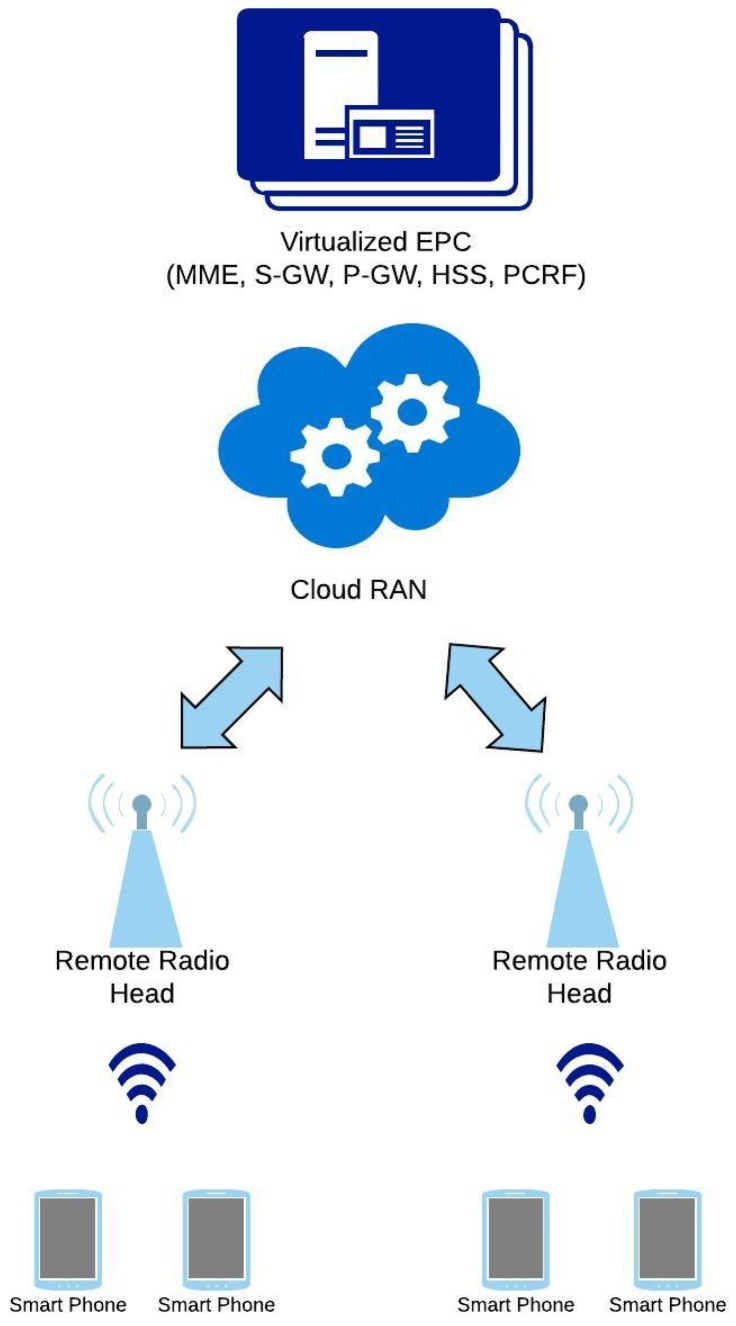
A ’virtualized’ 5G architecture.

Virtualization has been a very sought out way of reducing the time to market for the newer mobile technologies but with the emerging technological trends it might be a very useful way forward for reducing the energy consumption. In this case, hardware would serve as a bare metal for running multiple applications simultaneously for saving up on the cost of additional deployments of dedicated hardware and software components [48]. Most of the functions previously deployed on dedicated hardware would now be rolling out as software defined network functions thus promising scalability, performance maximization and mobility with in the cellular network. The virtual network architecture described in [50] lays out the interconnection between several virtual as well as the physical units being interconnected to form a larger system. A generalized 5G architecture incorporating virtualization has been illustrated in Figure 6. The smooth integration of different technologies with virtualized environment thus becomes the key of reaping the expected efficiency outcomes. Resource and operations management plays a vital role in actively regulating the system for a fine tuned state of execution that helps mitigate issues including redundancy and keeping the operating expenses under control. Furthermore, usage of an openflow switch would come in handy for efficient packet traversal within the network. Significant advantage in terms of reduced energy consumption of about 30% have been experienced by incorporating the current architecture with Network Function Virtualization (NFV). Authors have assumed an ideal case scenario that the virtual BBU will not consume any energy when it stays idle and also the advantage of the enormous computational pool in the form of cloud have been used.

Authors in [49] presented the significant energy conservation advantages of having virtual nodes in both access as well as the core network instead of having the physical nodes for executing only a single function. The proposed topology suggests baseband pooling for higher performance in the cloud, a direct gigabit optical connection from the remote radio heads to the core network and an even distribution of the core network nodes. The nearest available core network node would then be the one responsible of serving the incoming requests from the respective radio heads. The proposed architecture boasts the flexibility of resource distribution by having a single node running multiple virtualized access/core network functions e.g., serving gateway, packet gateway, etc. and the readiness of activating these functions wherever needed based on the work load. A visible gain of about 22% was recorded using mixed integer linear programming for modelling the work load across the nodes and both the core and access network were virtualized. Apart from the EE gains, a higher performance would also be achieved because of a reduced distance between the node requesting and the node serving the request. Research in [51] extends the same idea where the EE gains are deemed to be higher with an increased number of virtual function deployments in the access network which typically consumes more energy, about 70% of the entire demand of the end to end network. The suggested topology entails gigabit optical connectivity as the fronthaul technology instead of the Common Public Radio Interface (CPRI) connection between radio and baseband units. This brings out more deployment opportunities for the virtual machines by having more active nodes closer to the user. Authors documented a gain of about 19% with the proposed architecture. According to the authors in [52], existing RAN architecture needs modification for meeting the upcoming traffic demands. Baseband unit has been decomposed into two main parts, namely distributed unit and a central unit. Both units find their optimal placements either close to the users for serving the low latency demands or in remote areas for providing a pool of computational power. Mobile edge computing uses the same concept and NFV proves to be an enabling technology to use it to its full potential. The network layout comprises upon active antenna units and the central office for edge and access computation. Mobile edge computing units were housed along with the distributed and the central units and was the aggregator for the traffic. Both latter functions were virtualized on general purpose processors and finally the electronic switch was responsible for the traffic routing. Simulations conducted on this topology have revealed about 20% power saving as compared to the case of fixed deployment of hardware units. Moreover, Reference [53] also supports the idea of flexible centralization of RAN functions of small cells. Prominent outcomes would comprise upon interference mitigation in a dense deployment and reduced radio access processing. Authors in [54] devised an analytical model for calculating the optimal number of active operator’s resources. Dynamic Auto Scaling Algorithm, or DASA, was envisioned to provide a way for operators to better understand their cost vs performance trade off and authors have thus used real life data from Facebook’s data center for a realistic estimation. On top of the already established legacy infrastructure comprising mainly upon mobile management entity, serving gateway, packet gateway and the policy & charging function, 3GPP has now proposed specifications for a virtualized packet core providing on demand computational resources for catering to the massive incoming user requests. A comparison was drawn between the consumed power and the response time of the servers for the jobs in a queue by varying different factors including total number of virtual network function (VNF) instances, total number of servers available as well as the rate of the incoming jobs, total system capacity and the virtual machine (VM) setup times. Trends recorded from the plots have signified the saturation point of the system and have paved a way for operators to optimize their infrastructure to be robust without taking in more power than needed. Similarly [55] extends the above mentioned approach by taking into account the rejection of incoming requests in case the saturation point has been reached. A more realistic framework was presented that incorporates either dropping the jobs from the queue or even blocking them out from being registered until some resources could be freed up.

## 5. Review of SDN Technology for Enhancing EE

### 5.1. Energy Monitoring and Management in 5G with Integrated Fronthaul and Backhaul

**Figure 7:**
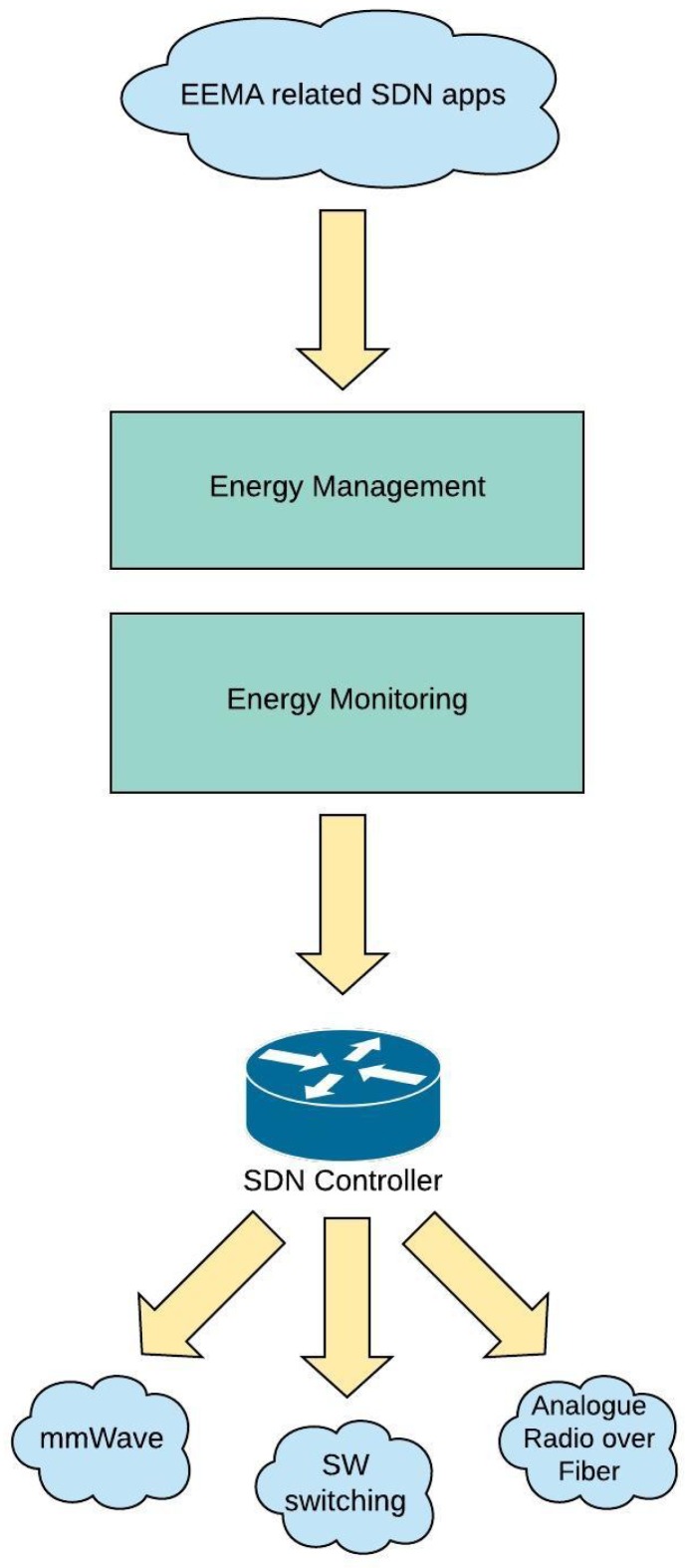
Energy Monitoring and Management via software defined networking (SDN).

The impact of software defined networking (SDN) on energy-efficiency was explored in [56]. The tremendous increase in the user density in a given area not only demands an energy efficient hardware but also demands for certain modifications in the control plane. Energy Management and Monitoring Applications (EMMA) were designed for observing the energy consumption in fronthaul as well as the backhaul network constituents. A monitoring layer was implemented over an SDN controller which observes the underlying operational domains including mmWave links and analogue Radio over Fiber technology (RoF). This topology is shown in Figure 7. The energy management framework was extended to provide analysis on virtual network slices as well by gathering the real time power consumption data of a server by a power meter installed with it and then incorporating it with the respective flows. EMMA is based upon a SDN/NFV integrated transport network using a Beryllium framework and supports features including energy monitoring of the access network and the optimization of power states for the nodes. Furthermore, an analytics module provide statistics on the traffic consumption by the currently ongoing services, Provisioning manager would help in setting up new network connections and dynamic routing of connections for the ongoing sessions based upon the energy aware routing algorithms. Authors have envisioned EMMA as a fronthaul technology for providing coverage for high speed trains. It comprises upon a context information module for collection of data for mobility, a statistics module for storing the contextual data and updating it regularly, and lastly the management module for consuming this data and making real time moves in the network by switching on the nodes as the train approaches and switching them off when it leaves. Significant energy savings ranging between 10 to 60% were demonstrated using the real life data by switching on the nodes exactly when needed and keeping them asleep otherwise [56].

### 5.2. Utility of Sleep Mode Energy Savings

In [57], authors discussed about getting benefited from the separated control and data planes in a heterogeneous network. Since this concept was not used in the previous generation of networks, further exploitation of this feature is expected to yield significant energy reductions. It was proposed that control plane communication would be done via low frequency macro cells and data plane information exchange would take place through high frequency femto cells. Detailed statistics about the daily traffic load and information about the kind of base stations deployed were tabulated. The application of the regular cell switch off technique especially in the off peak hours would yield reduced energy consumption of up to 48%. On the other hand, incorporation of power modulation at the femto cells would keep them operational and would yield energy savings of up to 27%. If the extreme isolation of control and data plane could be relieved then the macro cell would be able to serve the users with not just the control signaling but also with data transfer at low frequencies, resulting in a higher percentage of energy savings in the network. In addition to this concept, Reference [64] discusses the possibility of achieving 50–80% energy savings by incorporation of the energy aware heuristic algorithms.

## 6. Machine Learning Techniques for Energy-Efficiency in 5G

**Figure 8:**
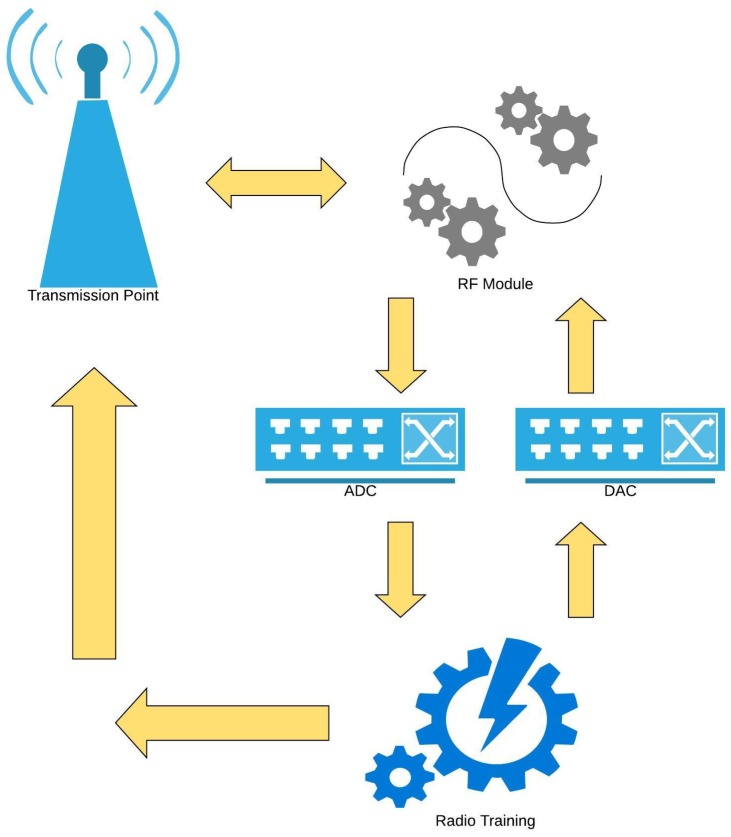
Dissection of a smart antenna.

Recently, machine learning techniques have been employed to various areas of wireless networks including approaches to enhance energy efficiency of the wireless network [58]. A typical example would include a smart transmission point, such as the one shown in Figure 8 that would evolve itself overtime by its observations.

In [59], the authors proposed switch-on/off policies for energy harvesting small cells through distributed Q-learning. A two tier network architecture was presented for discussion on on-off switching schemes based upon reinforcement learning. It is assumed that small cells are equipped to get their associated macrocell to transfer its load over to them and they themselves would rely upon the harvested energy, for example, solar energy. Application of Q-learning enables them to learn about the incoming traffic requests over time so they could tweak their operation to an optimal level. The proposed scenario includes a macro cell running on electricity and small cells running on solar energy with a distributed Q learning technique being used to gain knowledge about the current radio resource policies. Reward function for the online Q-learning proposes to turn off the small cells if users experience higher drop rates or use the ones that would already be on to take the burden from the macro cell. On the other hand, authors in [60] devised a novel EE and E2E delay duty cycle control scheme for controllers at the gateway of cellular and capillary networks. Formulation of a duty cycle control problem with joint-optimization of energy consumption and E2E delay was addressed followed by the distributed duty cycle control scheme.

In [61], the authors highlighted a distributed power control for two tier femtocell networks with QoS provisioning based on q-learning. Power control in the downlink of the two tier femtocell network was discussed and an effective network capacity measure was introduced for incorporating the statistical delay. Self-organization of small cells was also discussed with the perspective of Q-learning and utilization of a non cooperative game theory [61]. The proposed system model involves a macro base station covering several femtocells in its vicinity, each of them serving their own set of users. Expressions for SINR for both macro and femto cell users were also documented [61]. For the consumer’s energy efficiency, Pareto optimization was opted for as compared to the traditional multi-user scenarios, focusing on a system level energy efficiency instead.

Meanwhile in [62], the deployment of macro and pico base stations were made similar to the above scenario. However, the random deployment of femto BS by consumers cause interference problems and cognitive radio technology was put together with these femto BS for an improved spectrum access. Spectrum sensing techniques provide benefits for UL transmission since the femto cells are power limited as compared to the macro cells. Detailed mathematical analysis for spectrum sensing techniques using both hard and soft decisions were demonstrated in [62]. Authors formulated objective functions in such a way that although they are computing optimal power allocation for the users, the whole scheme incorporates constraints for energy efficiency maximization. In [63], the authors also use machine learning techniques for energy-efficient resource allocation in 5G heterogeneous cloud radio access network. Cloud radio access networks are considered as a key enabler in upcoming 5G era by providing higher data rates and lower inter cell interference. It consists of both small cells and macro base stations for accommodating more users, providing them with superior quality of service and for enhancing coverage area respectively where resources are scheduled through a cloud RAN. A resource allocation scheme was put together with the aim of maximizing energy efficiency of UEs served by the radio heads while minimizing inter tier interference [63]. Available spectrum was divided into two resource blocks and assigned to different UE groups depending upon their location and QoS demands. A central controller interfaced with the baseband unit pool gets to learn about the network state through the interfaced macro base station and then take certain actions needed for energy efficiency optimization. Furthermore, compact state representation was utilized for approximating algorithm’s convergence. The resource block as well as the power allocation with respect to energy saving in the downlink channel of remote radio heads in accordance with the QoS constraints has also been documented. Since the given model depends upon the prior UE knowledge for it to make transitions for optimization, Q-learning was proposed to practically model the objectives and system specifications. The resource allocation is mainly carried out at the controller in the BBU pool and the control signalling is carried out via the X1 and S1 links. The hierarchy of UEs and RRHs operate under macro base station and convey their states to the controller.

## 7. Challenges and Open Issues

In accordance with the increase in the computational demand from the base stations, in the upcoming 5G networks, energy efficiency needs to be scaled up by 100–1000 times in contrast with the traditional 4G network [1]. Since the transmission ranges would have been scaled down due the dense small cell deployment, the energy efficiency evaluation will potentially revolve around the computational side as compared to the transmission side previously. Storage functions for local data caching should also be considered in this evaluation, since it would potentially be common in the forthcoming networks. Scheduling schemes should be enhanced to involve an optimal number of antennas and bandwidth for resource allocation. The trade-off between transmission and computational power should be optimized considering the effects of the kind of transmission technology involved. Software Defined Networking might be a potential fix for this issue, yet it needs further exploration. Moreover, authors in [65] proposed the intermediate delays from source to destination to be incorporated in the energy efficiency formulation for an even more realistic estimation.

Most of the ongoing research has been discussing energy efficiency from a lot of different perspectives but so far a unifying approach has not been reached. Green Touch project has taken such an initiative but more exploration is needed for a stronger understanding [2].

With the explosive small cell deployment, 5G network would be interference limited so orthogonal transmission techniques might not be practical. The framework of sequential fractional programming might be extended for energy efficiency optimization with affordable complexity as suggested in [9]. Random Matrix theory and stochastic geometry appear as suitable statistical models for evaluating the randomness within the wireless networks, but a thorough research on energy efficiency needs to be conducted employing these tools.

Finally, the avenue of self-learning mechanisms is still less explored. Since local caching has been considered a potential answer for reducing the load on backhaul networks, novel approaches including this consideration need to be developed.

## 8. Conclusions

In this paper, we provide a survey of the state-of-the-art in energy-efficiency efforts in 5G. These new studies touch on several novel paradigms such as new radio, NOMA, ML-driven techniques and cache-enabled networks. Although there are several studies surveying the literature, our paper provides a clear classification of the proposed techniques with in-depth comparison. The paper is expected to be a road map for researchers in this field.

## Figures and Tables

**Table 1:** Summary of surveyed works.

Optimization Scope	Problem Addressed	Citation
EE at the BS level	Dissection of a BS and figures for energy consumption	[1]
	Downlink Massive MIMO Systems: Achievable Sum Rates and Energy Efficiency Perspective for Future 5G Systems	[16]
	Energy Efficiency in massive MIMO based 5G networks: Opportunities and Challenges	[17]
	EE improvement by a Centralized BB processing design	[18]
	Analytical modelling of EE for a heterogeneous network	[19]
	Energy Efficiency Metrics for Heterogeneous Wireless Cellular Networks	[20]
	Incentive based sleeping mechanism for densely deployed femto cells	[21]
	Sector based switching technique	[22]
	On interdependence among transmit and consumed power of macro base station technologies	[23]
	Utilization of Nash product for maximizing cooperative EE	[24]
	Energy Efficiency in Wireless Networks via Fractional Programming Theory	[25]
	Energy efficiency maximization oriented resource allocation in 5G ultra-dense network: Centralized and distributed algorithms	[26]
	Comparison of Spectral and Energy Efficiency Metrics Using Measurements in a LTE-A Network	[27]
	Energy Management in LTE Networks	[28]
	Energy-efficient resource allocation scheduler with QoS aware supports for green LTE network	[29]
	Interference-area-based resource allocation for full-duplex communications	[30]
	A resource allocation method for D2D and small cellular users in HetNet	[31]
	Highly Energy-Efficient Resource Allocation in Power Telecommunication Network	[32]
	EE enhancement with RRC Connection Control for 5G New Radio (NR)	[6]
	Proactive caching based on the content popularity on small cells	[7]
	Cooperative Online Caching in Small Cell Networks with Limited Cache Size and Unknown Content Popularity	[33]
	Economical Energy Efficiency: An Advanced Performance Metric for 5G Systems	[34]
	Energy-efficient design for edge-caching wireless networks: When is coded-caching beneficial?	[35]
	Content caching in small cells with optimized UL and caching power	[36]
	An effective cooperative caching scheme for mobile P2P networks	[37]
	EE analysis of heterogeneous cache enabled 5G hyper cellular networks	[8]
EE at the network level	Motivation for infrastructure sharing based on current energy consumption figures	[2,38]
	Energy efficiency in 5G access networks: Small cell densification and high order sectorisation	[39]
	Energy-Efficient User Association and Beamforming for 5G Fog Radio Access Networks	[40]
	Global energy and spectral efficiency maximization in a shared noise-limited environment	[9]
	EE Resource Allocation in NOMA	[41]
	Concept and practical considerations of non-orthogonal multiple access (NOMA) for future radio access	[42]
	Optimum received power levels of UL NOMA signals for EE improvement	[43]
	Spectral efficient nonorthogonal multiple access schemes (NOMA vs RAMA)	[44]
	Non-Orthogonal Multiple Access: Achieving Sustainable Future Radio Access	[45]
	Mode Selection Between Index Coding and Superposition Coding in Cache-based NOMA Networks	[46]
	Use case of shared UE side distributed antenna System for indoor usage	[47]
	Optimized Energy Aware 5G Network Function Virtualization	[48]
	Energy Efficient Network Function Virtualization in 5G Networks	[49]
	Network Function Virtualization in 5G	[50]
	A Framework for Energy Efficient NFV in 5G Networks	[51]
	Energy efficient Placement of Baseband Functions and Mobile Edge Computing in 5G Networks	[52]
	Energy Efficiency Benefits of RAN-as-a-Service Concept for a Cloud-Based 5G Mobile Network Infrastructure	[53]
	Dynamic Auto Scaling Algorithm (DASA) for 5G Mobile Networks	[54]
	Design and Analysis of Deadline and Budget Constrained Autoscaling (DBCA) Algorithm for 5G Mobile Networks	[55]
EE using SDN technology	Impact of software defined networking (SDN) paradigm on EE	[56]
	EE gains from the separated control and data planes in a heterogeneous network	[57]
EE using ML techniques	Machine Learning Paradigms for Next-Generation Wireless Networks	[58]
	Switch-on/off policies for energy harvesting small cells through distributed Q-learning	[59]
	Duty cycle control with joint optimization of delay and energy efficiency for capillary machine-to-machine networks in 5G communication system	[60]
	Distributed power control for two tier femtocell networks with QoS provisioning based on Q-learning	[61]
	Spectrum sensing techniques using both hard and soft decisions	[62]
	EE resource allocation in 5G heterogeneous cloud radio access network	[63]
